# Daily Spatial Complete Soil Moisture Mapping Over Southeast China Using CYGNSS and MODIS Data

**DOI:** 10.3389/fdata.2021.777336

**Published:** 2022-02-15

**Authors:** Ting Yang, Zhigang Sun, Jundong Wang, Sen Li

**Affiliations:** ^1^CAS Engineering Laboratory for Yellow River Delta Modern Agriculture, Institute of Geographic Sciences and Natural Resources Research, Chinese Academy of Sciences, Beijing, China; ^2^Shandong Dongying Institute of Geographic Sciences, Dongying, China; ^3^Key Laboratory of Ecosystem Network Observation and Modeling, Institute of Geographic Sciences and Natural Resources Research, Chinese Academy of Sciences, Beijing, China; ^4^College of Resources and Environment, University of Chinese Academy of Sciences, Beijing, China; ^5^National Meteorological Center, China Meteorological Administration, Beijing, China

**Keywords:** soil moisture (SM), CYGNSS, MODIS, daily, fusion

## Abstract

Daily spatial complete soil moisture (SM) mapping is important for climatic, hydrological, and agricultural applications. The Cyclone Global Navigation Satellite System (CYGNSS) is the first constellation that utilizes the L band signal transmitted by the Global Navigation Satellite System (GNSS) satellites to measure SM. Since the CYGNSS points are discontinuously distributed with a relativity low density, limiting it to map continuous SM distributions with high accuracy. The Moderate-Resolution Imaging Spectroradiometer (MODIS) product (i.e., vegetation index [VI] and land surface temperature [LST]) provides more surface SM information than other optical remote sensing data with a relatively high spatial resolution. This study proposes a point-surface fusion method to fuse the CYGNSS and MODIS data for daily spatial complete SM retrieval. First, for CYGNSS data, the surface reflectivity (SR) is proposed as a proxy to evaluate its ability to estimate daily SM. Second, the LST output from the China Meteorological Administration Land Data Assimilation System (CLDAS, 0.0625° × 0.0625°) and MODIS LST (1 × 1 km) are fused to generate spatial complete and temporally continuous LST maps. An Enhanced Normalized Vegetation Supply Water Index (E-NVSWI) model is proposed to estimate SM derived from MODIS data at high spatial resolution. Finally, the final SM estimation model is constructed from the back-propagation artificial neural network (BP-ANN) fusing the CYGNSS point, E-NWSVI data, and ancillary data, and applied to get the daily continuous SM result over southeast China. The results show that the estimation SM are comparable and promising (*R* = 0.723, root mean squared error [RMSE] = 0.062 m^3^ m^−3^, and MAE = 0.040 m^3^ m^−3^ vs. *in situ, R* = 0.714, RMSE = 0.057 m^3^ m^−3^, and MAE = 0.039 m^3^ m^−3^ vs. CLDAS). The proposed algorithm contributes from two aspects: (1) validates the CYGNSS derived SM by taking advantage of the dense *in situ* networks over Southeast China; (2) provides a point-surface fusion model to combine the usage of CYGNSS and MODIS to generate the temporal and spatial complete SM. The proposed approach reveals significant potential to map daily spatial complete SM using CYGNSS and MODIS data at a regional scale.

## Introduction

Soil moisture (SM) has a significant impact on the earth's ecosystem by affecting the hydrological processes and climate changes. Additionally, it plays a vital role in land surface evapotranspiration, water migration, and the carbon cycle. Meanwhile, SM is a critical link between the precipitation into runoff and the atmosphere (Long et al., 2019; Naz et al., 2020). Therefore, spatial complete and temporal continuous SM products are importantly needed for such applications. This reveals the necessity to map and analyze daily complete SM information with high spatial resolution (i.e., 1 × 1 km) in the long term and over a large scale.

Remote sensing products for SM, such as the Advanced Scatterometer (ASCAT) (Wagner et al., 2013), Soil Moisture Active Passive (SMAP) (Entekhabi et al., 2010), and Soil Moisture and Ocean Salinity (SMOS) (Kerr et al., 2012), which have strong penetration of vegetation, clouds, and fog, and are sensitive to the effects of water on the dielectric constant of the soil. However, the spatial resolutions are low (≥9 km), largely limiting the hydrological and agricultural applications. Additionally, the revisit periods are long (2~3 days), with a large number of gaps in daily data. SM estimated from land surface models (LSMs) [i.e., the Global Land Data Assimilation System (GLDAS) and the China Meteorological Administration Land Data Assimilation System (CLDAS)] has been released for public use, with spatial completeness and temporal continuity (Teuling et al., 2009; Bi et al., 2016; Meng et al., 2017). However, these products are primarily designed for global or continental scale SM studies, thus with a relatively low spatial resolution (i.e., 0.25° × 0.25° for GLDAS and 0.0625° × 0.0625° for CLDAS). Overall, SM information is rarely available at adequate spatial and temporal scales using a single remote sensing method.

The Cyclone Global Navigation Satellite System (CYGNSS) mission, data of which are publicly available, launched into space in December 2016 with eight microsatellites. The CYGNSS was designed to measure ocean winds in the tropics while reflections observed from the satellites are also proved sensitive to land parameters (Chew and Small, 2018). Meanwhile, the CYGNSS can capture surface-reflected GNSS signals over the tropics with a fine spatial [~25 × 25 km (incoherent), ~0.6 × 6.6 km (coherent, theoretical minimum)], and temporal (2.8–7 h) resolution (Eroglu et al., 2019; Yang et al., 2020). With the high spatial-temporal resolution, CYGNSS provides a new technical way for large-scale surface SM retrieval. The University Corporation for Atmospheric Research (UCAR) first developed and published the sub-daily of the CYGNSS SM data product (Chew and Small, 2020a). However, this SM product is obtained using surface reflectivity (SR) and its correlation with SMAP SM, heavily relies on SMAP SM product, and has a relatively low spatial resolution (36 × 36 km).

Since the CYGNSS points are discontinuous with relativity low density, continuous SM cannot be mapped with a high accuracy using CYGNSS data alone. Simple interpolation, such as linear interpolation, is a widely used image-based gap-filling method (Kornelsen and Coulibaly, 2014; Cui et al., 2020). However, this method could not obtain a high-quality SM. Thus, the CYGNSS points should be combined with other continuous remotely sensed data to obtain a high-precision SM distribution. The Moderate-Resolution Imaging Spectroradiometer (MODIS) data can provide the spectral information of the soil surface related to the SM and have a finer spatial resolution than CYGNSS and passive remote sensing SM products (Babaeian et al., 2018). Some recent studies have proposed different multi-source remote sensing fusion methods for SM spatial reconstruction, e.g., Kalman filtering, triple collocation, random forest, and back-propagation (BP) neural network (Xu et al., 2018; Fu et al., 2019; Long et al., 2019; Kim et al., 2021; Wu et al., 2021). Nevertheless, it should be noted that the existing algorithms mostly ignore the missing data caused by the influence of the cloud on the optical remote sensing data, which lead to the discontinuity of the fusion result. Fortunately, multi-temporal reconstruction methods have been developed to recover the missing optical data for cloudy and foggy pixels (Long et al., 2019).

In this study, a point-surface fusion method is proposed integrating the CYGNSS points, MODIS data, CLDAS products, ancillary information, and *in situ* SM measurements using the BP artificial neural network (BP-ANN) model, to generate spatial complete and daily continuous 1 × 1 km SM. To achieve this objective, we: (1) match the CYGNSS surface reflectivity (SR) with dense *in situ* measurements, and evaluate its performance to estimate daily SM; (2) combine land surface temperature (LST) output from the CLDAS (0.0625° × 0.0625°) and MODIS (1 × 1 km) to generate spatial complete and temporal continuous LST maps, and propose the Enhanced Normalized Vegetation Supply Water Index model, hereafter, named the E-NVSWI model to estimate SM at high spatial resolution and spatial completeness; (3) fuse the two results with the ancillary data to establish point-surface fusion model using the BP-ANN, subsequently, use for mapping daily continuous SM result over Southeast China.

## Data Used

### CYGNSS Data

The CYGNSS level 1 data, science data record version 2.1 product is used in this study. The CYGNSS receivers process delay-Doppler maps (DDM) as the main observatory product. The DDM instruments are designed to map the scattered signal on the ocean and land surface, which is sampled in time and frequency, thus delivering DDMs at the proximity of the specular point (SP) with the surroundings (Clarizia et al., 2019; Chew and Small, 2020a). The signal-to-noise ratio (SNR) is the metadata derived from DDM, which is used to estimate the SR at SP, since an ideal SM retrieval approach of CYGNSS data product would acquire the SR approximated based on the bistatic radar equation. The associated information, e.g., the incidence angle, the noise, and the antenna gains, are included in the metadata.

The CYGNSS data preprocessing contained four steps: (1) the antenna gain greater than 0 dB (corresponding to uncertainties reported in the measured antenna gain patterns), (2) the elevation angle of the specular points higher than 30° (to keep the good-quality left hand circularly polarized (LHCP) data), (3) the data located in bare soil and low vegetated density regions (i.e., vegetation height <5 m) identified by the Global land cover map for 2009 (GlobCover 2009) are selected to exclude the effects of vegetation cover, buildings, inland water bodies, etc., and (4) the Quality Flags (i.e., direct signal in DDM and low confidence in the Global Positioning System effective isotropic radiated power [GPS EIRP] estimate) in the CYGNSS L1b data are used to select the good data acquisitions.

### MODIS Data

The MODIS/Aqua Land Surface Temperature and Emissivity (LST/E) Daily L3 Global 1 km Grid V006 (MYD11A1) dataset (1 km spatial resolution) is used in this study to derive daily night- and day-time LSTs. The final LST value used in this study is the average value of day and night LSTs values. In addition, vegetation index (VI) data from MODIS surface reflectance products 8-Day L3 Global (MOD09Q1) dataset (250 m spatial resolution) product, and albedo data from daily MCD43A3 are also obtained, respectively.

The MODIS data are processed based on the MODIS Reprojection Tool (Dwyer and Schmidt, 2006; Duan et al., 2017), and all these products are transformed and registered to the geographic coordinate system. The MOD09Q1 data and MCD43A3 data are then resampled to 1 km using the nearest neighbor resampling method.

### CLDAS Data

The China Meteorological Administration Land Data Assimilation System is a grid fusion product covering the Asian region (0–65° N, 60–160° E), with a spatial resolution of 0.0625° × 0.0625° and temporal resolution of 1 h (Meng et al., 2017). The CLDAS released the official products of forcing data from 2009. The dataset is generated from multiple sources (i.e., the *in situ* measurement and satellite products), and contains air temperature, pressure, LST, SM, precipitation, and solar radiation, etc. The CLDAS data are downloaded *via* http://data.cma.cn.

In this study, LST and SM from the CLDAS product are used as inputs of the algorithms to reconstruct the daily LST, and the final verification data, respectively. The CLDAS products from 0:00 to 23:00 are averaged in the present study.

### *In situ* Measurements

**Figure 1:**
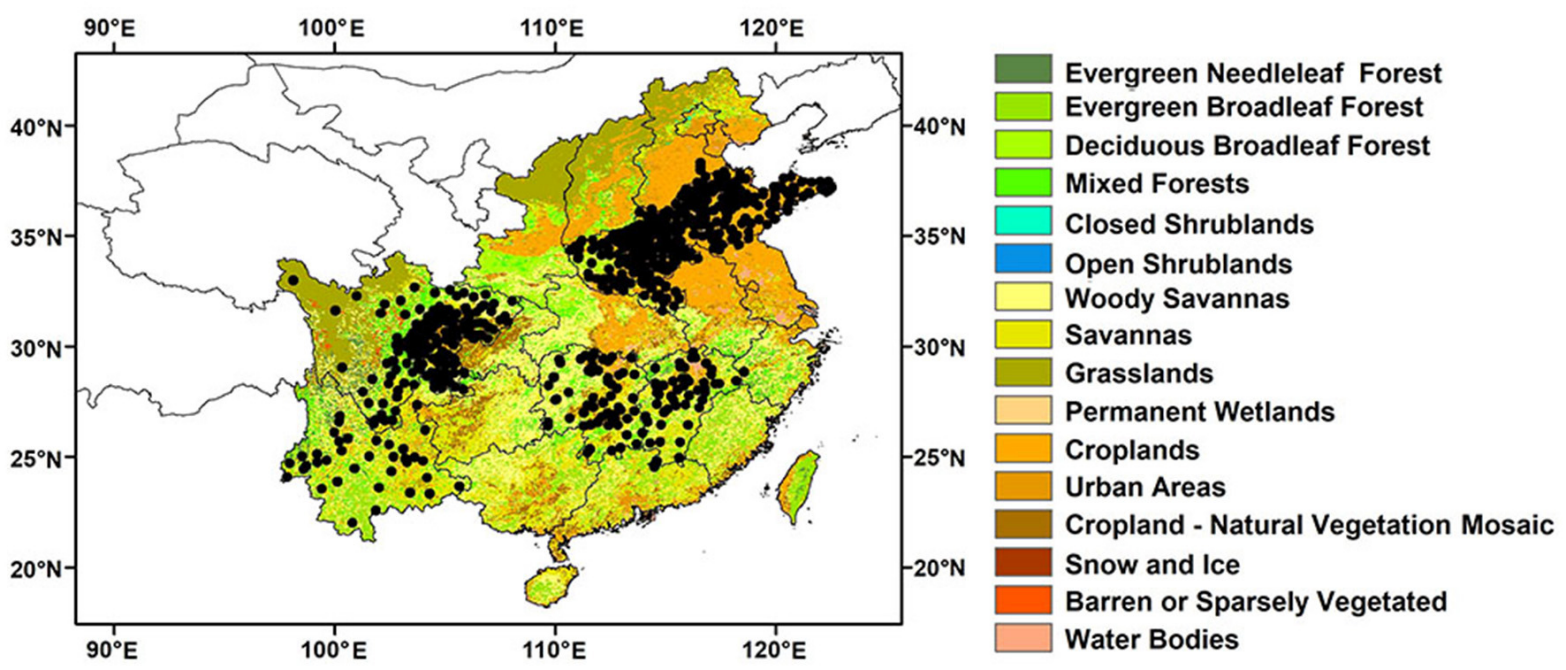
Locations of the 596 *in situ* SM sites in the southeast of China. The background map is the MODIS IGBP data set.

Daily averaged *in situ* SM data of 596 sites collected on day 1, 11, and 21 in March, April, July, August in 2018 are provided by the China Meteorological Administration (CMA) (Figure 1). The data are collected by the ASMO sensors at a depth of 10 cm. Considering the complex geographical environment and climate conditions in China, the selected sites are distributed in six provinces of China (i.e., Henan, Hunan, Shandong, Jiangxi, Sichuan, and Yunnan), with different land covers, climate conditions, and terrain distributions (Table 1). All the *in situ* SM data are collected. The background map is MODIS The International Geosphere-Biosphere Program (IGBP) dataset. Table 2 is the summary of the datasets used in this study.

**Table 1:** Characteristics of the *in situ* measurements.

**Province**	**Numbers of sites**	**DEM value (m)**	**Climate condition**	**Land cover**
Henan	194	20–2,318	Temperate continental monsoon	Cropland Deciduous Broadleaf Forest Savannas Urban Areas
Sichuan	178	192–6,457	Subtropical monsoon	Evergreen Broadleaf Forest Woody Savannas Cropland - Natural Vegetation Mosaic
Yunnan	36	81–5,929	Tropical monsoon/ Plateau mountain	Evergreen Broadleaf Forest Savannas Croplands
Hunan	60	9–1,993	Subtropical monsoon	Evergreen Broadleaf Forest Woody Savannas Cropland - Natural Vegetation Mosaic
Jiangxi	49	6–2,093	Subtropical humid	Evergreen Needleleaf Forest Evergreen Broadleaf Forest Woody Savannas Cropland - Natural Vegetation Mosaic
Shandong	79	39–1,451	Temperate monsoon	Croplands Grasslands

**Table 2:** Summary of the datasets used in this study.

**Dataset**	**Temporal resolution**	**Spatial resolution**
CYGNSS L1	Daily	0.6–6.6 km
MODIS LST(MYD11A1)	Daily	1 km
CLDAS LST/SM	Daily	0.0625°
MODIS VI (MOD09A1)	8-day	500 m
MODIS Albedo MCD43A3	Daily	500 m
*In situ* data (0–10 cm)	Daily	/
GPM precipitation	Daily	3 km
SRTM DEM	/	90 m
SRTM Aspect	/	90 m
SRTM Slope	/	90 m

## Method

**Figure 2:**
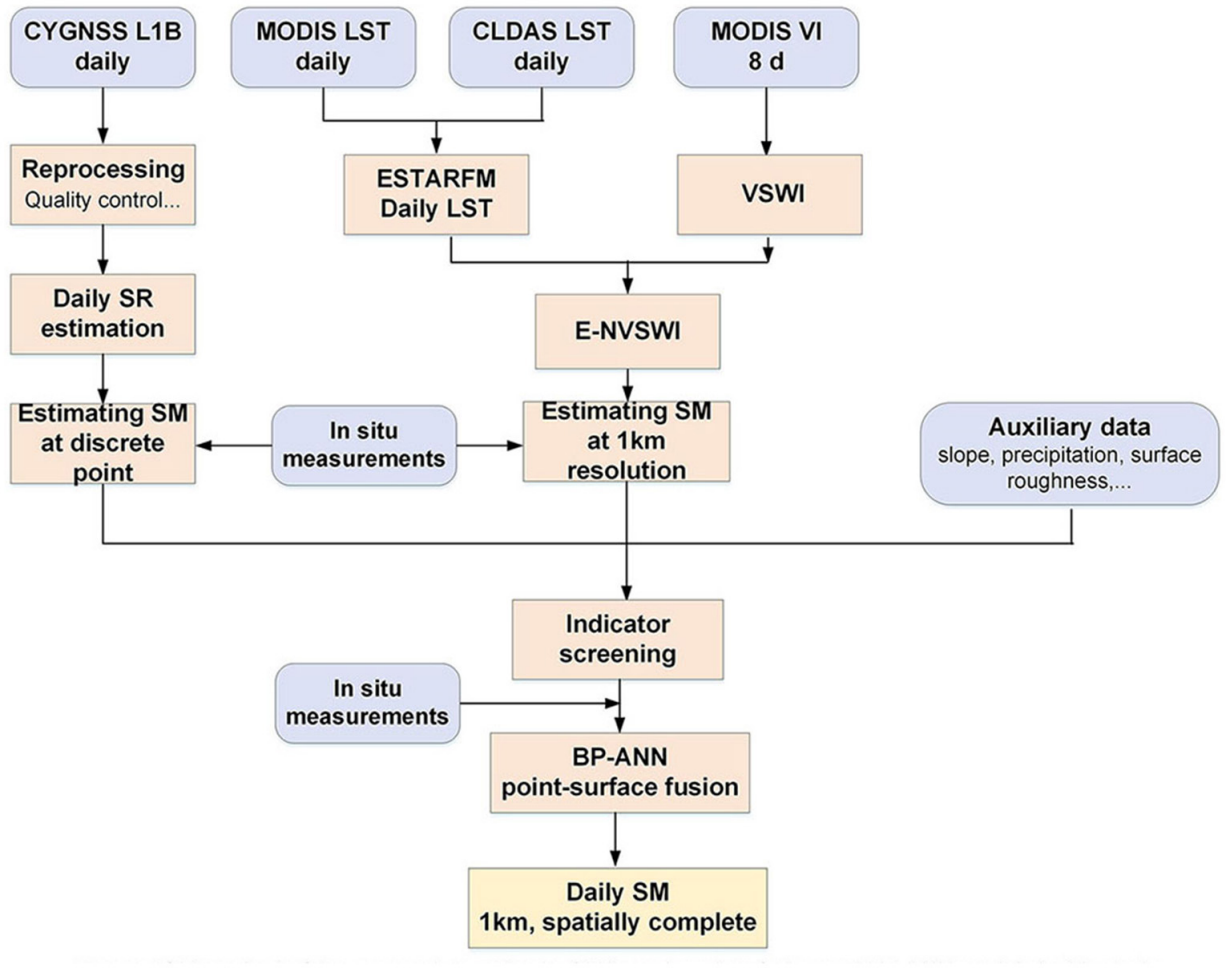
Flow chart of the approach to estimate SM based on data fusion and BP-ANN models in this study.

The methodology section consists of: (1) the SM estimation over CYGNSS points using CYGNSS derived SR and *in situ* measurements; (2) the LST reconstruction using the Enhanced Spatial and Temporal Adaptive Reflectance Fusion Model (ESTARFM) model, and E-NVSWI model development as the SM proxy using optical remote sensing data; (3) the CLDAS, MODIS, and auxiliary data fusion using the BP-ANN model, and the daily spatial complete SM mapping; and (4) evaluation results with *in situ* and CLDAS SM. The overall approach is summarized in the flowchart shown in Figure 2.

### SM Derived From CYGNSS

Surface reflectivity is the primary parameter in the SM retrieval algorithm of CYGNSS, since the peak value of each DDM mainly presents the SR and is sensitive to the changing SM values and surface conditions. So, in this study, SR is primarily characterized by the bistatic radar equations to acquire the SR. Moreover, the peak value of the DDM is affected by other variables unrelated to the reflecting surface, such as the incidence angle and distances from SP to the transmitter and receiver. The SR (in dB) can be described as follows (Chew and Small, 2020a,b):


(1) $$\begin{array}{l} SR=SNR-10\log P_{r}^{t}-10\log G^{t}-10\log G^{r}-20\log\lambda \\ +20\log\left(R_{ts}+R_{sr}\right)+20\log\left(4\pi \right) \end{array}$$

where SNR is the peak power minus the noise N, $P_{r}^{t}$ is the transmitted power, *G*^*t*^ is the gain of the transmitting antenna, *G*^*r*^ is the antenna gain toward the specular reflection point, and λ represents the wavelength of the GPS L1 bands signal, *R*_*ts*_ is the range from the transmitter to the specular reflection point, *R*_*sr*_ is the range from the specular reflection point to the receiver, and is the incidence angle.

**Figure 3:**
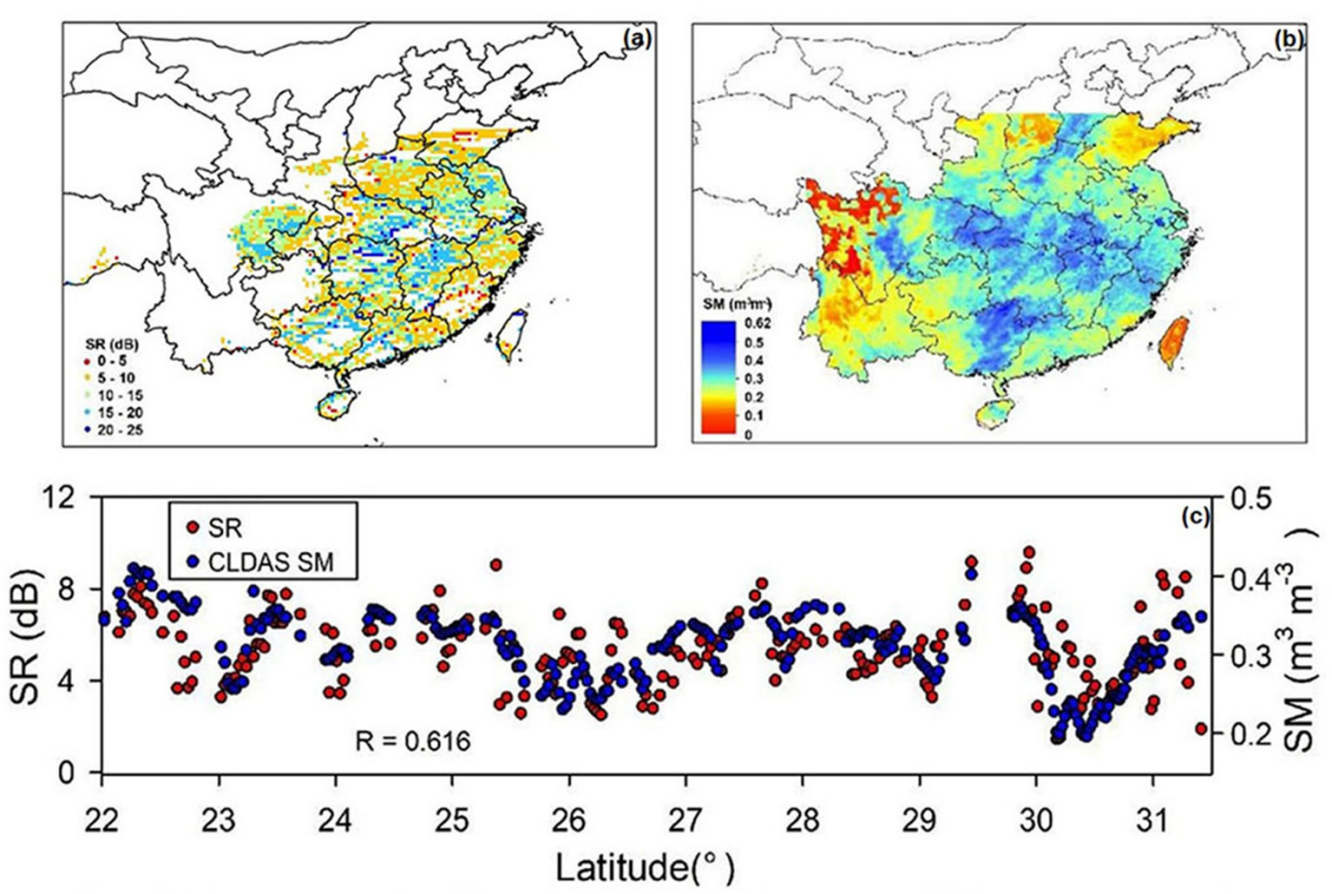
**(a)** Distribution of CYGNSS SR on April 21, 2018, **(b)** Distribution of CLDAS SM on April 21, 2018, and **(c)** Comparisons of the SR derived from CYGNSS against CLDAS SM for locations with latitudes between 22° to 31.5°.

The UCAR developed the CYGNSS SM data product using SR and its correlation with SMAP SM, which led to heavy reliance on SMAP SM products. Previous studies have shown that the SMAP may underestimate in vegetation-disturbed areas primarily as a result of biased surface temperature data (Fan et al., 2020). Subsequently, the UCAR SM product may also transmit the biases. Additionally, the product is gridded with a relatively coarse spatial resolution of 36 × 36 km. In contrast to the UCAR SM product, the SM on the CYGNSS point in this paper is estimated using *in situ* measurements. Figures 3a,b show the distribution of CYGNSS SR against the GLADS SM on April 21, 2018. Overall, the SR can well reflect the SM dynamics during the observed period. Spatially, the estimated SR varies significantly. Figure 3c is the result of the comparisons of the CYGNSS SR against CLDAS SM for the location with latitudes between 22 and 31.5°. The correlation coefficient (*R*) between SR derived from CYGNSS and CLDAS SM data is 0.616.

**Figure 4:**
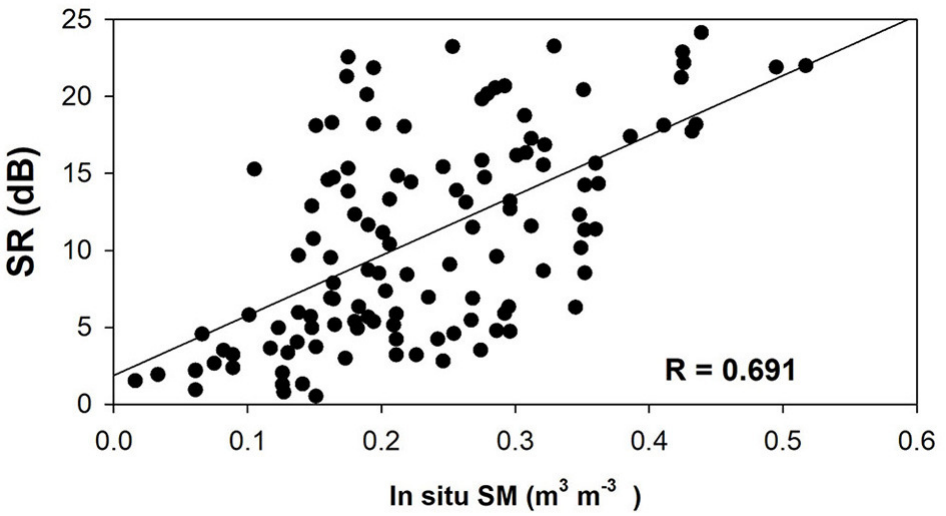
Comparisons of SR and *in situ* SM.

For each *in situ* site shown in Figure 1 during the entire observation period, the CYGNSS observations located less than 0.5 km from this site are selected. Additionally, the values of SR are normalized to 0–25 dB overall, to the product values in a range that intuitively makes sense. Then, the linear regression between SR and *in situ* SM match-ups is calculated. The linear regression between SR and *in situ* SM match-ups is calculated, the R for the relationship is shown in Figure 4. The data of both CYGNSS and *in situ* are selected from March to July 2018. The segmented calculation equation of SM derived from CYGNSS is:


(2) $$\begin{array}{l} \{\begin{array}{c} SM_{CYGNSS}=0.0011\times SR+0.0352\;\left(SR<5dB\right)\\ SM_{CYGNSS}=0.0089\times SR+0.1336\;\left(SR>5dB\right) \end{array} \end{array}$$

### SM Derived From MODIS Data

#### LST Reconstruction Using ESTARFM Model

The ESTARFM is used to generate spatio-temporally consistent LST. The ESTARFM produces a synthetic MODIS-like (1 × 1 km) image from a CLDAS (0.0625° × 0.0625°) input image on the prediction date (Zhu et al., 2010; Long et al., 2019). Two pairs of cloud-free MODIS and CLDAS images should be input in the first step. The effective pixels of MODIS need to account for more than 85%. Then, based on these shoulder pairs, the statistical relationship between MODIS and CLDAS is first established with ESTARFM model using linear regression. Finally, the resulting linear regression models are used to translate the coarse and spatial complete CLDAS data to reconstructed LST changes.

**Figure 5:**
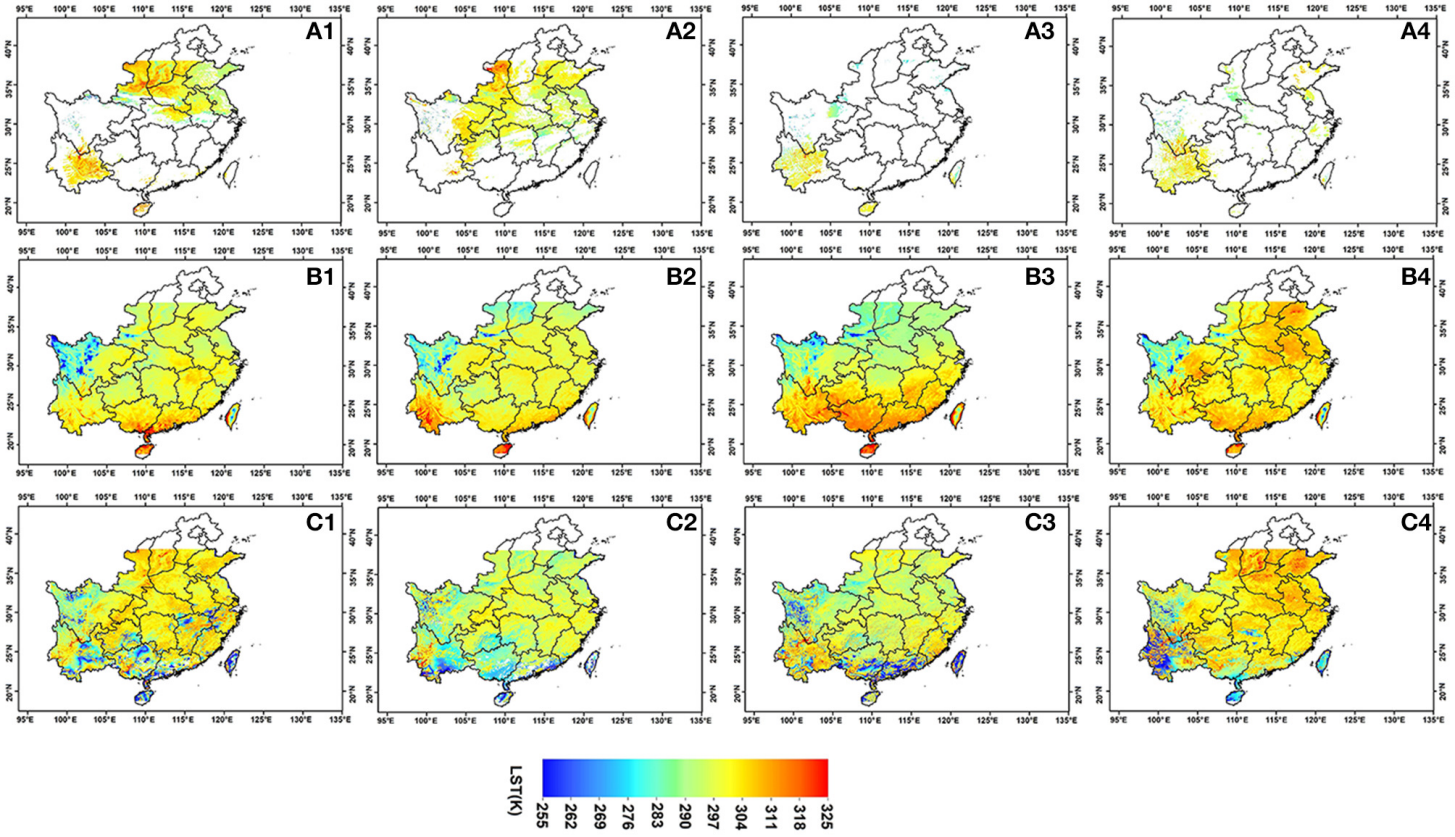
Spatial comparisons of MODIS LST, CLDAS LOT and reconstructed LST **(A1–A4)** are MODIS LST in April 7, April 14, April 21, and April 28 in 2018, **(B1–B4)** are CLDAS LST in April 7, April 14, April 21, and April 28 in 2018, **(C1–C4)** are reconstructed LST in April 7, April 14, April 21, and April 28 in 2018.

Figure 5 shows the spatial comparisons of MODIS LST, CLDAS LST, and reconstructed LST. The reconstructed LST can provide more details and spatial complete and temporally continuous LST information at MODIS resolution, which provides a critical input for SM estimating to be illustrated in the following sections.

#### E-NVSWI From MODIS as SM Proxy

The vegetation index and LST combined usage method is widely used to estimate SM in optical remote sensing. NVSWI based on the division of normalized difference vegetation index (NDVI) and LST has been proven to be useful in estimating regional SM. Result accuracy is better than other methods, e.g., the TVDI method (Cong et al., 2017). The NVSWI can represent not only the relative spatial location but also the comparison of the time series. NVSWI is most commonly calculated as:


(3) $$\begin{array}{l} NVSWI=\frac{\left(VI/LST\right)-{\left(VI/LST\right)}_{min}}{{\left(VI/LST\right)}_{max}-{\left(VI/LST\right)}_{min}} \end{array}$$

where VI is the vegetation index, LST is the land surface temperature, (*VI*/*LST*)_min_ and (*VI*/*LST*)_max_ are the minimum and maximum ratio values of the pixel during the period of study, respectively,

In previous studies, NDVI is the most used VI in NVSWI. However, studies have shown that NDVI is sensitive to the chlorophyll pigment in plants, which may lead to a better performance at high-vegetation periods and a low correlation at low-vegetation periods. Additionally, VSWI_min_ and VSWI_max_ are calculated from the whole region. However, a large-sized sampling window helps to increase pixel heterogeneity but also results in variation in the sampling window.

In this study, an E-NVSWI model is proposed to solve the aforementioned problems. First, the Modified Soil Adjusted Vegetation Index (MSAVI) is more advantageous than NDVI in describing the bare soil line and the vegetation coverage in the soil background (Zhang et al., 2014). Therefore, the MSAVI is used in this study instead of NDVI to calculate the E-NVSWI. The MSAVI can be described as:


(4) $$\begin{array}{l} MSAVI=0.5*(2*DN_{band1}+1\\ -\sqrt{{\left(2*DN_{band2}+1\right)}^{2}-8*(DN_{band2}-DN_{band1})}) \end{array}$$

Second, a suitable range is proposed for defining an ideal VI–LST diagram. The range can be considered as the upper limit of the sampling window size. The most optimal sampling window size is chosen as 10 × 10 km, after times of testing.

**Figure 6:**
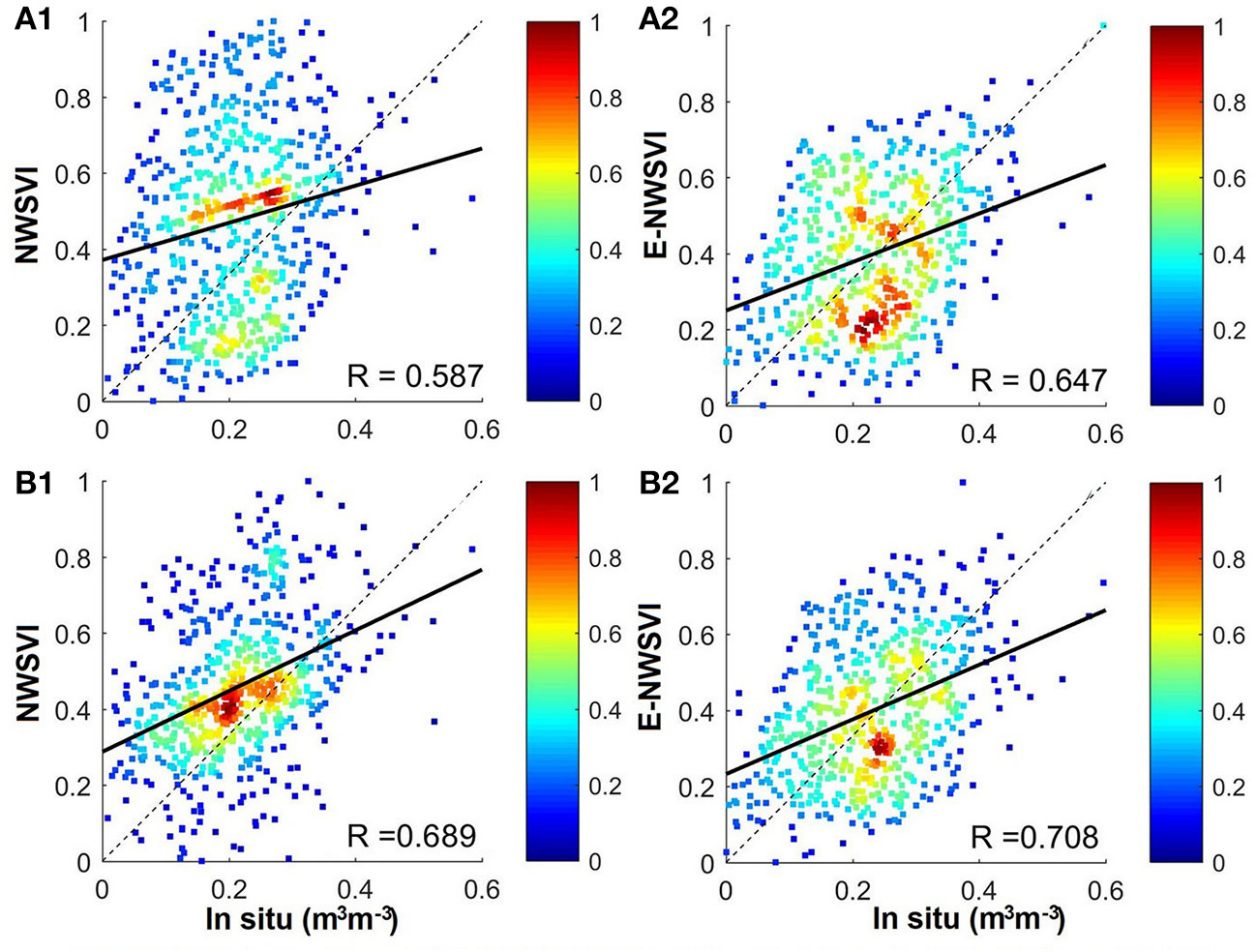
Comparision of NWSVI/E-NWSVI with *in situ* SM on March 1, 2018 and April 1, 2018, **(A)** March 1, 2018; **(B)** April 1, 2018.

Figure 6 shows the density plots of the NVSWI and E-NVSWI versus the *in situ* SM on March 1, 2018, and April 1, 2018, respectively. The NVSWI and E-NVSWI are normalized between 0 and 1 to show a visual consistency, which does not affect the final accuracy. Overall, E-NVSWI better reflects the SM dynamics during the observed period, and shows a better agreement with *in situ* SM (*R* varies from 0.649 to 0.708 vs. *R* varies from 0.587 to 0.689).

### Indicator Screening of Input Parameters and Structures for the ANN Model

The geophysical parameters such as vegetation, topography, air temperature, and precipitation have all been proved to affect SM to some extent (Murphy et al., 2009; Eroglu et al., 2019; Yang et al., 2020). On the other hand, the physical basis of using CYGNSS for SM monitoring is that the L band is highly sensitive to the changes in soil dielectric constant, which is mainly with respect to the presence of SM. Meanwhile, the confounding factors of vegetation (i.e., vegetation water content [VWC]) and surface roughness would reduce the sensitivity of the L band to SM. Thus, the utilization of these ancillary data is necessary. In addition to E-NWSVI, six auxiliary variables representing the abovementioned parameters are also considered (i.e., precipitation, VWC, digital elevation model [DEM], slope, aspect, surface roughness, slope, and air temperature). Then, a new model considering the aforementioned variables is constructed using the BP-ANN to estimate continuous SM over the study area.

The VWC and roughness are computed from NDVI and slope with empirical relations as follows (Jackson et al., 1999; Campbell et al., 2018; Eroglu et al., 2019),


(5) $$\begin{array}{l} VWC=\left(1.9134\times NDVI^{2}-0.3215\times NDVI\right)\\ +stemfactor\times \frac{NDVI_{max}-NDVI_{\min}}{1-NDVI_{min}} \end{array}$$

(6) $$\begin{array}{l} Roughness=\frac{C^{2}}{\text{cos}\left(DEM_{slope}\times^{\pi }{/}_{180}\right)} \end{array}$$

Where stemfactor is the parameter from a land cover-based lookup table (LUT), and C is the cell size.

Before establishing the BP-ANN SM estimation model, the mean impact values (MIVs) are used to choose the variables. Table 3 lists the degree ranking of nine variables with their MIVs on April 1, 2018. The output layer is *in situ* measurements. The variables are chosen when they have an accumulative of at least 98%. Seven variables: E-NWSVI, VWC, slope, aspect, DEM, surface roughness, and precipitation accounted for 98.45% of the cumulative MIV contribution and are subsequently further used.

**Table 3:** Indicator screening for BP-ANN SM model.

**Variables**	**MIV**	**Accumulative contribution**
E-NWSVI	0.213	30.93%
VWC	0.154	53.29%
Slope	0.078	64.61%
Aspect	0.085	76.96%
DEM	0.041	82.91%
Surface roughness	0.085	95.25%
Precipitation	0.022	98.45%
Albedo	0.0088	99.72%
Air temperature	0.0019	100.00%

### BP-ANN Method

The BP-ANN is a supervised learning algorithm, which referred to a multi-layers forward neural network with an input layer, one or more hidden layers, and an output layer, structurally. The main idea of the BP-ANN regression task is to establish the nonlinear function between the input layers and output layer. BP-ANN can feasibly add more related samples and variables, not limited in parametric, and is widely used for downscaling microwave SM products (Yang et al., 2018; Cui et al., 2020).

**Figure 7:**
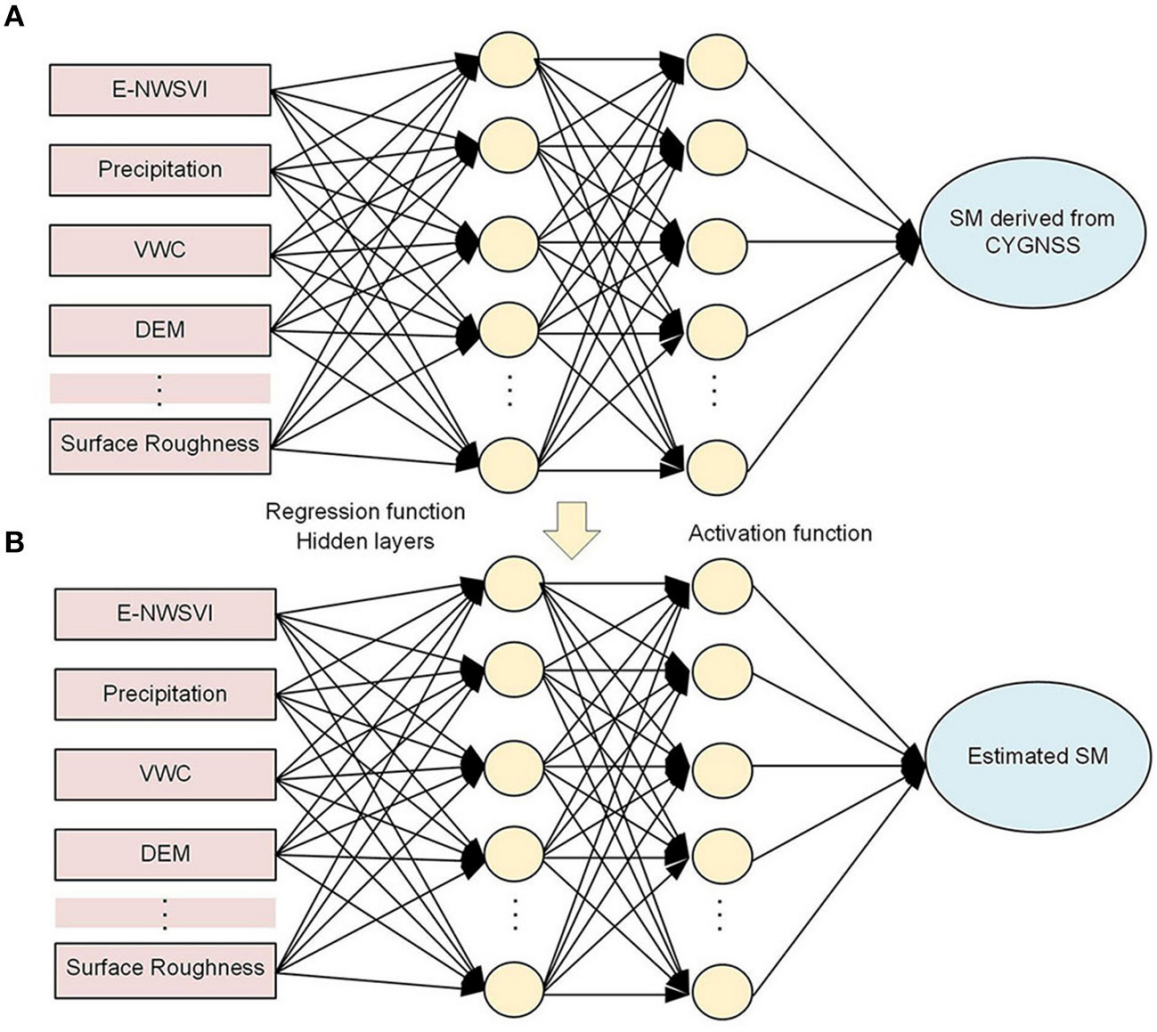
The BP-ANN model to estimate daily SM. **(A)** Training. **(B)** Testing.

The CYGNSS reflectivity is not only sensitive to SM, but also sensitive to other geophysical parameters, e.g., vegetation canopy, elevation, slope, surface roughness, and precipitation. Thus, for daily SM estimates, multifactor non-linear regression BP-ANN considering the aforementioned variables is constructed to estimate continuous SM over the study area (Figure 7). In the training stage, the input layer contains seven nodes which are “surface scale” data with continuous distribution in terms of E-NWSVI, precipitation, VWC, DEM, etc. The output layer is the CYGNSS SM, which is calculated using the *in situ* SM measurements combined with equation (2), and in the form of scattered points. Then, a non-linear relationship can be constructed between the surface and point scale data. Thus, in the testing stage, the non-linear relationship can be used to estimate the continuous SM. Data from March to August 2017 are used as the training dataset, and data from March to August 2018 are used as the testing dataset.

The training set is used to adjust the weights on the neural network, and the testing set is used for testing the network performance. The datasets are normalized to obtain values between 0 and 1 prior to the training. Repeated training sets are tested to obtain an optimal neural network structure to achieve reasonable results. The ANN structure used in this paper is as follows: the input layer has six nodes, which is the same as the number of used features. The input parameter is E-NWSVI, VWC, albedo, DEM, surface roughness, and precipitation, respectively. The output layer has a single node representing the SM derived from CYGNSS in the training stage, and the SM values in the testing stage, respectively. There are three hidden layers, and the number of nodes is seven. The hyperbolic tangent is chosen as the activation function. The last layer is a regression layer with no activation function. The maximum train number is set to 6,000, the error metric is being minimized as root mean squared error (RMSE), the error threshold is set to 0.001, and the learning rate is set to 0.05.

The study area is southeast China. When training the model, each input layer is “surface scale” data with a continuous distribution, and the number of valid pixels is 4,780,129. The number of points in the output layer depends on the number of CYGNSS per day, varying from 62,332 to 824,13.

## Results

### Spatial Distributions

**Figure 8:**
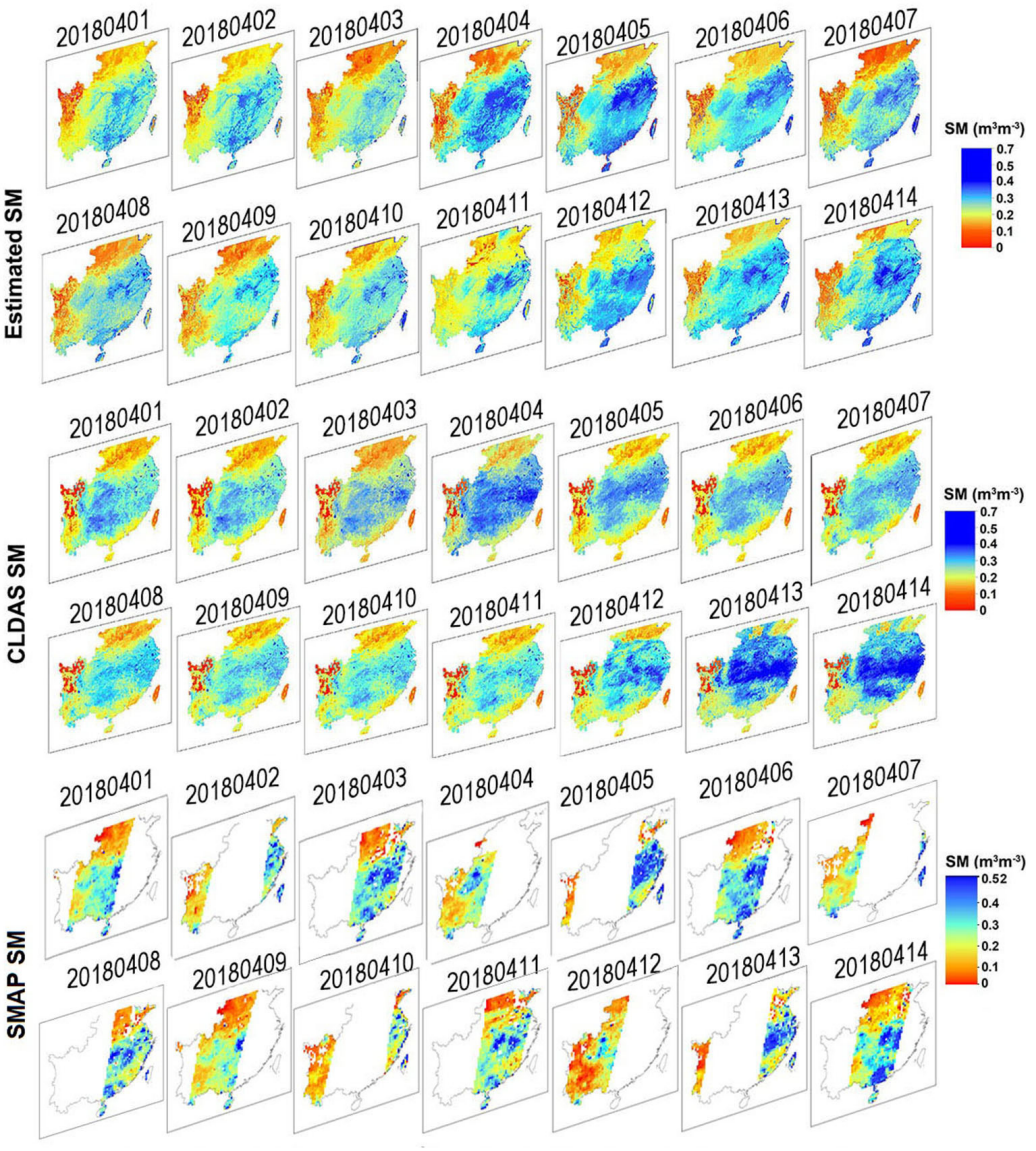
Spatial comparisions of the estimated SM (1 × 1 km). CLDAS SM, SMAP SM on fourteen consecutive days from April 1 to April 14, 2018.

Figure 8 compares the estimated SM, CLDAS SM, and SMAP SM on 14 consecutive days from April 1 to April 14, 2018 (using the R, RMSE, and mean absolute error (MAE) as indicators). Here, it should be noted that the CLDAS LST and CLDAS SM are different products. The CLDAS LST is obtained by integrating the ground station data and atmospheric driving products and the near real-time. The CLDAS SM is comprised mainly based on Ensemble Kalman Filter (EnKF) and land process models integrating precipitation of atmospheric forcing data and surface-incident solar radiation data received from hourly outputs of the FY2 geostationary meteorological satellite, and observation data (Zeng et al., 2021). The spatial resolution of SMAP SM grid is 36 km. Due to the design of satellite orbits, gaps exist in daily SMAP SM products provided by microwave sensors. The MAE, RMSE, and R of the estimated SM vs. CLDAS SM during the 14 days varied from 0.046–0.051 m^3^ m^−3^, 0.050–0.069 m^3^ m^−3^, and 0.607–0.735, respectively. The MAE, RMSE, and R of the estimated SM vs. SMAP SM during the 14 days varied from 0.042–0.049 m^3^ m^−3^, 0.044–0.061 m^3^ m^−3^, and 0.629–0.757, respectively. Overall, the results indicate that the BP-ANN models can produce spatial complete and temporally continuous daily SM of high accuracy. In addition, the estimated SM showed similar spatial patterns with the CLDAS SM and the SMAP SM. This is mainly attributed to the SM estimated from CYGNSS providing high-precision SM information. The SM showed an increasing trend, particularly in the central study region. Furthermore, SM values on some days are higher than those on other days, mostly due to precipitation that maintained the SM at higher values, particularly on April 4 and 5, 2018.

It is apparent that more abundant information of the estimated SM is presented than that of the CLDAS SM on the same day. Compared with the CLDAS SM, the estimated SM shows good performance and more detailed information on spatial characteristics, e.g., the western part of the region. It should be noted that, there are apparent mistakes in CLDAS SM simulations, with Taiwan showing quite low levels throughout April. Since the CLDAS SM is simulated based on the land process models, driving data, and data assimilation method, the complex calculation process may lead to this phenomenon.

**Figure 9:**
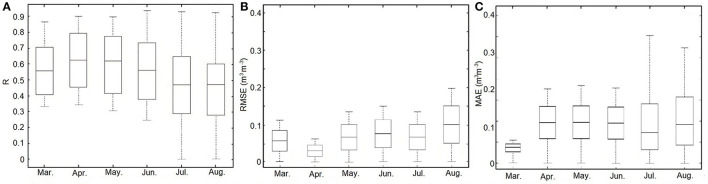
Boxplot of three statistical indices (i.e., R, RMSE, and MAE) between the estimated SM and *in situ* SM. **(A)** R, **(B)** RMSE, and **(C)** MAE.

Figure 9 displays the numerical distributions of three statistical indices (i.e., R, RMSE, and MAE) of performances at a daily scale for the estimated SM compared with *in situ* measurements from March to August in 2018. Generally, all the performances of the three indices are poorer during the period from June to August than other months. Specifically, in Figure 9A, R reaches its highest value in April and obtains its lowest value in August. From June to August, the R values exhibit a decreasing trend. The variation of RMSE is contrary to that of R, which means that a higher R-value always indicates a lower RMSE value (Figure 9B). It is clear that the RMSE in April is the lowest in the observed period. As for the variations of MAE (Figure 9C), it shows similar trends as RMSE. Incoherent scattering due to volume scattering from dense vegetation from June to August could be the possible reason for this phenomenon.

### Temporal Distributions

**Figure 10:**
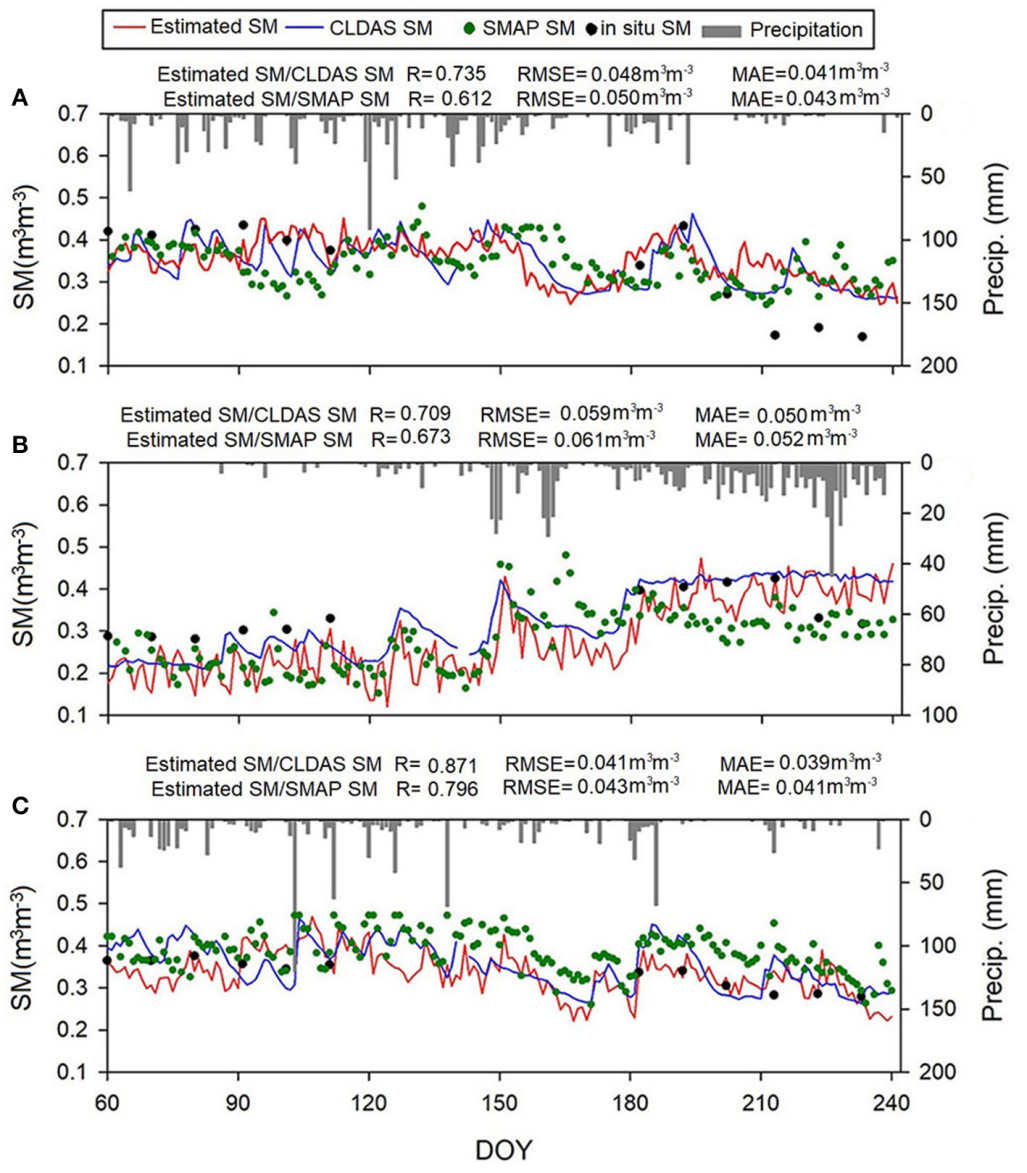
Temporal variations in the estimated SM, CLDAS, and *in situ* SM at three validation sites, **(A)** is Hunan, **(B)** is Jiangxi, and **(C)** is Yunnan.

Three sites in Hunan, Jiangxi, and Yunnan, representing different climate conditions and vegetation densities, are chosen randomly to further analyze the temporal variations of the estimated SM from the day of year (DOY) 60 to 240. Figures 10A–C shows the time series at each site. The synchronous ground precipitation data are also provided for comparison purposes. All of the estimated SM time series generally capture temporal variations in CLDAS SM and SMAP SM and showed good temporal consistency with the CLDAS SM and SMAP SM at different locations (R varies from 0.709 to 0.871 vs. CLDAS SM, R varies from 0.612 to 796 vs. SMAP SM). Several peaks are shown in Figure 10 during the observation period, which is consistent with the variations of precipitation events. Note that in Figures 10A,C, relatively low SM values mainly appear in the harvest periods of June and October without irrigation. Oppositely, the SM at the Jiangxi site appears upward trend with the monthly growth. The rainy season in this site may lead to this phenomenon.

**Figure 11:**
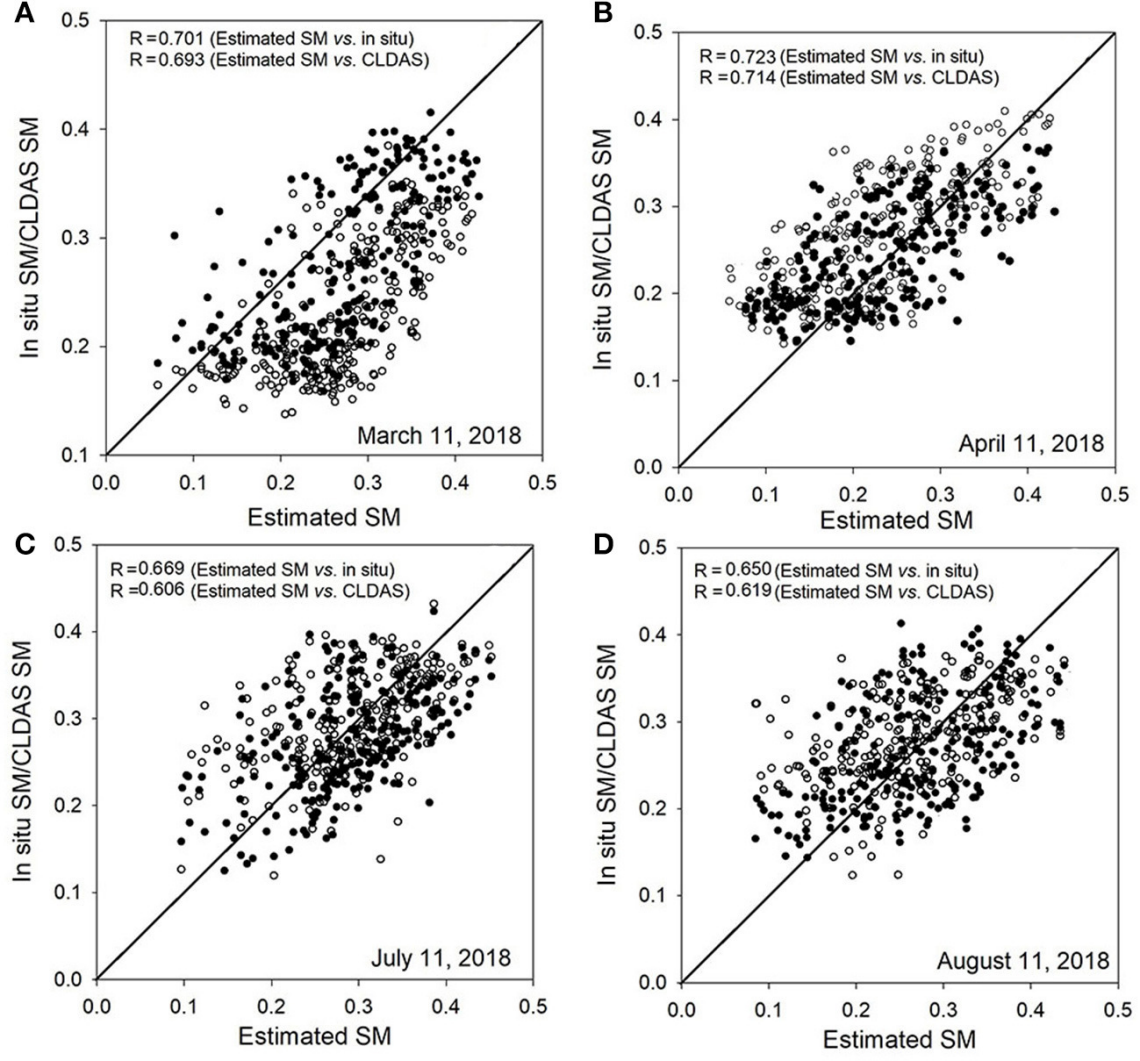
Validations of estimated SM against CLDAS and *in situ* SM at four days in 2018 over southeast Mainland China. The solid point represents estimated sm vs. *in situ* SM, and the hollow point represents estimated sm vs. CLDAS SM. **(A)** March 11, 2018, **(B)** April 11, 2018, **(C)** July 11, 2018, and **(D)** August 11, 2018.

To further evaluate the performances of the estimated SM, the estimated SM is validated separately against CLDAS SM and *in situ* SM at 4 typical days in 2018 (i.e., March 11, April 11, July 11, and August 11). Figures 11A–D shows the validation results, respectively. Table 4 shows the statistical indices of the four days. In general, the R values are more than 0.65, and the RMSE and MAE values are less than 0.062 and 0.040 m^3^ m^−3^ over the entire period time, respectively. In addition, according to the validation results, the statistical indices of estimated SM against CLDAS SM outperform the indices of estimated SM against *in situ* SM. In terms of MAE, the results of estimated SM against CLDAS SM are also lower than those of estimated SM against *in situ* SM.

**Table 4:** Statistical indices for the China Meteorological Administration Land Data Assimilation System (CLDAS) SM, *in situ* soil moisture (SM) and estimated SM.

**Data type**	**Indices**	**March, 11**	**April, 11**	**July, 11**	**August, 11**
*In situ* SM	R	0.701	0.723	0.669	0.650
	RMSE (m^3^m^−3^)	0.059	0.062	0.057	0.051
	MAE (m^3^m^−3^)	0.038	0.040	0.024	0.023
CLDAS SM	R	0.693	0.714	0.606	0.619
	RMSE (m^3^m^−3^)	0.054	0.057	0.059	0.052
	MAE (m^3^m^−3^)	0.025	0.039	0.027	0.024

## Discussion

### Comparison With Other Methods

To obtain daily SM data, many studies have focused on developing algorithms using multi-source remote sensing data. Table 5 shows a summary of relevant studies using multi-source remote sensing data to estimate daily SM. From Table 5, it can be concluded that our advantages exist in: (1) for the first time ever, provides a point-surface fusion model to combine the usage of CYGNSS and MODIS to generate the temporal and spatial complete SM; and (2) produce spatial complete and daily continuous 1 × 1 km SM in the southeast China with a comparable result and even slightly better result with the previous studies.

**Table 5:** Summary of relevant studies to obtain daily SM data using multi-source remote sensing data.

**Publication**	**Data used**	**Spatial resolution**	**Objectives**	**Key results**
Abbaszadeh et al. (2019)	MODIS SMAP Precipitation, topography, *In situ* SM	1 km	Downscales the level 3 daily SMAP SM at 1-km spatial resolution over clear-sky	(1) R varies from 0.44 to 0.86 (2) RMSE varies from 0.015 to 0.065 m^3^m^−3^
Hongtao et al. (2019)	SMAP *In situ* SM	9 km	Extends the SMAP 9-km SM by developing a non-local filter based on STFM Model	(1) R varies from 0.7 to 0.9 (2) RMSE varies from 0.052 to 0.101 m^3^m^−3^
Long et al. (2019)	MODIS ESA CCI CLDAS GLDAS *In situ* SM	1 km	Generates spatially complete and daily continuous SM	(1) R varies from 0.64 to 0.72 (2) RMSE varies from 0.050 to 0.063 m^3^m^−3^
Liu et al. (2020)	MODIS DEM ECV SM *In situ* SM	1 km	Validates the performance of multiple machine learning algorithms in downscaling the ECV SM dataset	(1) R varies from 0.2 to 0.713 (2) RMSE varies from 0.053 to 0.076 m^3^m^−3^

### Advantages and Limitations

In this study, the satellite data (CYGNSS, MODIS, and ASTER DEM), model simulation data (i.e., CLDAS), and *in situ* measurements are integrated to build models to estimate the daily SM. The results showed that the model could be successfully applied to produce spatial complete and daily continuous 1 × 1 km SM in the southeast of Mainland China. First, for CYGNSS data, the results show it can estimate SM with accuracy comparable with CLDAS. Moreover, as the first GNSS-R constellation, CYGNSS can provide detailed spatial variabilities of SM with a very short revisit time. The GNSS-R payload is light in weight and cost-effective, which makes it possible to design small satellite constellations. Second, LST is reconstructed to reduce the impact of clouds on MODIS remote sensing data. Thus, an important variable with full spatial coverage is provided in reflecting spatial and temporal variability in SM. Third, the E-NVSWI model can consider the pixel heterogeneity and variation in the VI and LST in the sampling window and improve the accuracy to estimate SM with full spatial coverage than the NVSWI model. Finally, the BP-ANN is used to fuse the point-surface multi-sources. The final estimated results can well reflect temporal variability and spatial heterogeneity in SM, which demonstrated that BP-ANN can be a potential method to solve the classification problem or fusion of point and surface heterogeneity.

The estimated SM results showed a temporal and spatial change consistent with the CLDAS SM products, and *in situ* SM. However, it is necessary to investigate the error source of the SM retrievals. Possible limitations of this study may be the following: (1) different spatial scales between CYGNSS points, optical remote sensing pixels, and the CLDAS data. The spatial resolution of CYGNSS (~0.6–6.6 km) is quite different from that of the *in situ* measurements (~0.0025 m^2^), meaning that data inconsistency exists between the different products. Although *in situ* measurements from dense sites are used to address the issue, the differences in spatial resolution continue to reduce the deviations. In addition, since the CLDAS pixel with a relatively coarse spatial resolution, simple downsampling methods may lead to the lack of detailed information on CLDAS. (2) Difficulty in matching different remote sensing datasets to each other in daily values. Since the daily MODIS VI and LST datasets are severely affected by cloud and fog, for VI applications, the 8 days of synthetic data are used.

## Conclusion

This article explored the feasibility of generating daily spatial complete SM mapping over Southeast China using CYGNSS and MODIS data. The results indicated that combining the CYGNSS data and MODIS data, the daily spatial complete SM with a relatively high accuracy can be mapped over southeast China (R = 0.723, RMSE= 0.062 m^3^ m^−3^, and MAE = 0.040 m^3^ m^−3^ vs. *in situ*, R = 0.714, RMSE= 0.057 m^3^ m^−3^, and MAE = 0.039 m^3^ m^−3^ vs. CLDAS).

The *in situ*, model, and satellite data are integrated to estimate the SM dataset, which leverages the advantages of every single product. The result will meet the need for daily continuous monitoring of SM for land surface evapotranspiration and water resources management. Our future work will focus on improving the accuracy of the public SM product, such as calibrating the attenuation of vegetation and surface roughness and downscaling its spatial resolution. Additionally, since land cover may have a significant impact on the SM of a location, future research may use the land surface type to cluster the data first and try other machine learning methods (i.e., random forest) to continue the research.

## Data Availability Statement

The original contributions presented in the study are included in the article/supplementary material, further inquiries can be directed to the corresponding author/s.

## Author Contributions

TY and ZS: conceptualization and writing, reviewing and editing, and funding acquisition. TY and JW: methodology. SL: resources. TY: writing the original draft preparation. ZS: supervision. All authors contributed to the article and approved the submitted version.

## Funding

This study is jointly supported by the Strategic Priority Research Program of the Chinese Academy of Sciences (XDA23050102 and XDA19040303), the Key Project of the Chinese Academy of Sciences (KJZD-SW-113), and the National Natural Science Foundation of China Projects (Grant No. 42101376).

## Conflict of Interest

The authors declare that the research was conducted in the absence of any commercial or financial relationships that could be construed as a potential conflict of interest.

## Publisher's Note

All claims expressed in this article are solely those of the authors and do not necessarily represent those of their affiliated organizations, or those of the publisher, the editors and the reviewers. Any product that may be evaluated in this article, or claim that may be made by its manufacturer, is not guaranteed or endorsed by the publisher.
